# THE MORE THINGS CHANGE, THE MORE THEY STAY THE SAME: CHANGE AND CONTINUITY IN ENDURING SOCIAL TIES

**DOI:** 10.1093/geroni/igac059.482

**Published:** 2022-12-20

**Authors:** Athena Koumoutzis, Kelly Cichy

## Abstract

Enduring social ties with family and friends hold important implications for health and well-being across adulthood. Social relationships are simultaneously sources of support and strain, and both positive and negative aspects of relationships change daily and over time. This symposium explores continuity and change in relationship strains (i.e., conflicts, support needs) experienced in the context of enduring social relationships, particularly in response to anticipated and emerging needs for support in later life. First, Meinertz, Gilligan, and Suitor use qualitative data from spousal dyads to compare mothers’ and fathers’ explanations of which adult child they prefer as their future caregiver. Next, using longitudinal data across two waves, Bui, Kim, and Fingerman investigate how different types of past support exchanges between parents and adult children are associated with older parents’ care receipt and expectations. Third, Koumoutzis, Cichy, and Kinney explore the association between change in parental disability and adult children’s intergenerational ambivalence (i.e., both positive and negative sentiments), including the extent to which adult children’s stress and reward appraisals mediate the link between parental disability and ambivalence. Kyungmin and colleagues explore how older adults (age 62-76) felt burden in their relationship with their very old parents (age 81-101) and what factors are associated with feelings of burden across two cultural contexts, the U.S. and Korea. Lastly, using ecological momentary assessment, Birditt and colleagues examine longitudinal trajectories of negative ties (i.e., irritating, demanding) and the links between daily positive and negative social interactions and emotional well-being.

